# Rescue endoscopic ultrasound-guided gastroenterostomy with closing of the duodenum in case of duodenal perforation

**DOI:** 10.1055/a-2479-1311

**Published:** 2024-12-04

**Authors:** Meddy Dalex, Mariola Marx, Elodie Romailler, Sébastien Godat

**Affiliations:** 1 30635 Division of Gastroenterology and Hepatology, Lausanne University Hospital, Lausanne, Switzerland

A duodenal perforation is a life-threatening condition 1 with a mortality rate between 8% to 25% 2 . We will present two cases of duodenal perforations managed endoscopically.

Our first case is a 74-year-old man admitted for ulcer-induced peritonitis after nonsteroidal ani-inflammatory drug ingestion. An abdominal computed tomography (CT) scan showed a pneumoperitoneum with periduodenal infiltration. A laparoscopic exploration confirmed peritonitis without duodenal perforation. Two days later, the patient presented with a resurgence of abdominal pain, prompting an upper endoscopy, which found a necrotic cavity at the first part of the duodenum treated with a 12-cm fully covered duodenal prosthesis. The clinical evolution was unfavorable with the persistence of the duodenal perforation. We decided to perform an endoscopic ultrasound (EUS)-guided gastrojejunostomy with a lumen-apposing metal stent (LAMS) (Hot AXIOS 20 mm; Boston Scientific, Marlborough, Massachusetts, USA). The pylorus was closed in the same session with an over-the-scope (OTS) clip (Ovesco Endoscopy AG, Tübingen, Germany). The patient was discharged after 2 weeks after a rapid recovery ( Video 1 , Fig. 1 ).

**Fig. 1:**
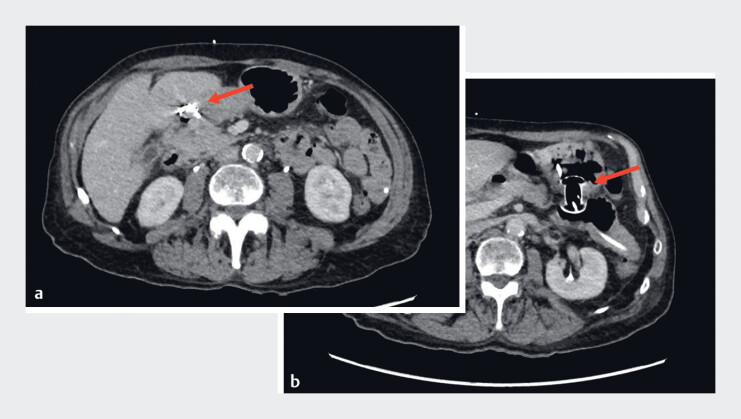
Abdominal computed tomography. **a** Metal prosthesis closing the pylorus in the duodenum. **b** Gastro-entero-anastomosis with lumen-apposing metal stent.

Gastro-entero-anastomosis with lumen-apposing metal stent and pylorus closing.Video 1

Our second case is a 54-year-old man admitted to the intensive care unit for severe acute Balthazar E pancreatitis with multi-organ failure, a duodenal perforation in communication with a large necrotic retroperitoneal cavity, extra-hepatic bile ducts destruction, and a pancreatic fracture. Two EUS-guided cystogastrostomies with LAMS (Hot AXIOS 10 mm; Boston Scientific) were done to treat paragastric collections. The biliary defect was treated with a fully covered metallic stent. After one month, the retroperitoneal cavity increased in size with the resurgence of sepsis. An EUS-guided gastrojejunostomy with LAMS (Hot AXIOS 20 mm; Boston Scientific) was performed, with closing of the genu superius with an OTS clip (Ovesco Endoscopy AG) and positioning an Endo-SPONGE (B. Braun, Melsungen, Germany) into the duodenal bulb. Progress was excellent with a discharge from acute care and complete removal of the Endo-SPONGE after 2 weeks.

These two successful EUS-guided gastro-entero-anastomoses with LAMS and endoscopic closing of the duodenum open up a new alternative when duodenal perforation occurs and cannot be treated surgically.

Endoscopy_UCTN_Code_TTT_1AO_2AI

## Notes

Endoscopy E-Videos https://eref.thieme.de/e-videos
*E-Videos* is an open access online section of the journal *Endoscopy* , reporting on interesting cases and new techniques in gastroenterological endoscopy. All papers include a high-quality video and are published with a Creative Commons CC-BY license. Endoscopy E-Videos qualify for HINARI discounts and waivers and eligibility is automatically checked during the submission process. We grant 100% waivers to articles whose corresponding authors are based in Group A countries and 50% waivers to those who are based in Group B countries as classified by Research4Life (see: https://www.research4life.org/access/eligibility/ ). This section has its own submission website at https://mc.manuscriptcentral.com/e-videos .
